# Single-cell RNA sequencing: enhancing the predictive accuracy of tumor immunotherapy efficacy

**DOI:** 10.1042/EBC20253017

**Published:** 2025-08-22

**Authors:** Wei Zhou, Ziwei Huang, Zhiyun Wu, Mengyuan Tang, Linqi Zhu, Weifeng Shi, Qi Wang, Liangzhu Feng

**Affiliations:** 1 Department of Clinical Laboratory, The Third Affiliated Hospital of Soochow University, ChangZhou, Jiangsu, 213003, China; 2 Department of Biological Treatment, The Third Affiliated Hospital of Soochow University, ChangZhou, Jiangsu, 213003, China; 3 Institute of Functional Nano & Soft Materials (FUNSOM), Jiangsu Key Laboratory for Carbon-Based Functional Materials & Devices, Soochow University, Suzhou, Jiangsu, 215123, China

**Keywords:** biomaterials, immune checkpoint blockade therapy, immunotherapy, single-cell RNA sequencing, tumor microenvironment

## Abstract

The swift advancement of single-cell RNA sequencing (scRNA-seq) technology has furnished a crucial instrument for investigating the tumor microenvironment (TME) and its response to immunotherapy. As immunotherapy becomes increasingly prevalent, the challenge of accurately predicting its efficacy has emerged as a prominent focus in contemporary research. In recent years, the utilization of scRNA-seq in the context of immunotherapy has demonstrated promising potential, particularly in the realms of efficacy prediction and biomarker discovery. The heterogeneity of immune cells within the TME exerts intricate and multifaceted influences on treatment response, necessitating comprehensive investigation. Furthermore, the integration of biomaterials into tumor immunotherapy presents novel research opportunities in this domain. scRNA-seq technology offers a systematic approach to evaluating the modifications in the TME induced by biomaterials. This article aims to review the current state of scRNA-seq in the context of immunotherapy, identify existing challenges within related research, and propose future research directions.

## Introduction

Tumors represent intricate assemblies comprising neoplastic cells, immune cells, and stromal cells, characterized by both intratumoral and intertumoral heterogeneity. Immunotherapy, an innovative therapeutic approach, including immune checkpoint blockade (ICB) therapy, adoptive cell transferring therapy, and others, has been developed to eradicate tumor cells by harnessing the patient’s immune system. Considering the host cells within the tumor microenvironment (TME) like cancer-associated fibroblasts (CAFs), tumor-associated macrophages (TAMs), and regulatory T cells (Tregs) can secrete a range of immunosuppressive factors to impede anti-tumor immune responses [1–3], it has been uncovered that the TME significantly impairs the therapeutic potency of immunotherapy and many other cancer treatments. Recently, based on their varying response efficacy to immune checkpoint inhibitors and the degree of T cell infiltration, the solid tumors can be categorized into four distinct phenotypes: immune hot, immune cold, immunosuppressive, and immune rejection. Clinical evidence has indicated that only these ‘immune hot’ tumors distinguished by elevated T cell infiltration, increased PD-L1 expression, a high tumor mutation burden (TMB), and enhanced interferon-γ (IFN-γ) signaling exhibit favorable responses to ICB therapy, the most prevalently applied immunotherapy in clinics [4,5]. Even though immunotherapy has made revolutionary progress, a large quantity of patients still cannot benefit from it. It is therefore urgent to identify appropriate biomarkers to precisely predict the immunotherapeutic efficacy of immunotherapy in clinical applications and avoid unnecessary toxicity. To date, biomarkers including PD-L1 expression, microsatellite instability-high (MSI-H), and TMB have been validated to be effective for predicting the therapeutic efficacy of ICB therapies. However, given that the limited throughput of these detection methods cannot provide a comprehensive characterization of the TME status [6], developing high-throughput detection methods is therefore highly desired to enable more precise prediction of the therapy efficacy of immunotherapy.

Recently, with the emergence of the next-generation sequencing technology that enables the rapid sequencing of millions of DNA or RNA fragments simultaneously, comprehensively analyzing the genomic signatures of specific cell subtypes has been confirmed to be a promising tool to benefit the precision medicine approaches. Among them, single-cell RNA sequencing (scRNA-seq) provides a powerful means to unravel the complexity and heterogeneity of the transcriptome at the individual cell level. Compared with the bulk RNA sequencing, scRNA-seq offers superior insights into cellular states and functions, which would benefit the identification of unique cell type characteristics at the omics level and the investigation of intercellular interactions and cellular changes under specific physiological or pathological conditions. Therefore, it has been extensively explored for evaluating the complexity and heterogeneity of the TME, aiming at predicting the tumor progression and the efficacy of immunotherapeutic interventions [7,8]. Till now, there are 79 registered cancer treatment clinical trials utilizing scRNA-seq to identify tumor-specific molecular markers and cell subgroups, explore the differences in TME composition, provide a basis for diagnosis and precision treatment, build a cellular atlas of whole tumor biomarkers for targeted cancer treatments, or explore the efficacy and safety of ICB (Table 1) [9–15].

**Table 1:** Registered clinical trials utilizing scRNA-seq to identify effective predictive indicators for the efficacy of anti-tumor immunotherapy

ClinicalTrials.gov ID	Area	Cancer type	Purpose of scRNA-seq
NCT05361564	Republic of Korea	NSCLC (non-small cell lung cancer)	Identify molecular mechanism of DTP causing innate drug resistance to neoadjuvant brigatinib in resectable NSCLC harboring ALK fusion by analyzing scRNA-seq
NCT05056896	Belgium	CRC (colorectal cancer)	Assess the difference in the change of ISC marker using scRNA-seq data analysis
NCT04312308	Location not provided	NSCLC (non-small cell lung cancer)	Explore early biomarker to predict response and overall survival after atezolizumab therapy by scRNA-seq
NCT06241092	China	Papillary thyroid carcinoma	scRNA-seq was employed to capture genetic alterations and TME heterogeneity
NCT04603248	Republic of Korea	HNSCC (head and neck squamous cell carcinoma)	Explore the biomarkers to predict objective response by scRNA-seq
NCT04162691	China	Thymoma	Find out how it affects genetic and protein expression in patients with malignant thymoma by scRNA-seq
NCT05056896	United States	Colorectal cancer	Assess the difference in the change of ISC marker using scRNA-seq
NCT06495749	China	Pancreatic cancer	Analyzing RNA expression in peripheral blood mononuclear cell using scRNA-seq
NCT06022341	France	Secondary leukemia, myeloproliferative neoplasm	Identified pathways among the different subgroups by integration using scRNA-seq
NCT04434833	China	Non-Hodgkin lymphoma	Using scRNA-seq to explore the heterogeneity of lymphoma inside and outside the lymph node, identify tumor-specific molecular markers and cell subgroups, explore the differences in tumor microenvironment composition, and provide a basis for diagnosis and precision treatment
NCT05821790	Location not provided	Meningioma	Identify differential expression and gene ontology of expressed genes in meningioma cells, dura mater cells, and associated immune cells by using scRNA-seq and RNAscope analyses
NCT06407310	Location not provided	Triple-negative breast cancer	The molecular state of cells in the TME will be measured at a single-cell level using scRNA-seq before and after one dose of pembrolizumab
NCT05850273	France	Myeloproliferative neoplasm	The trajectories of hematopoietic differentiation will be analyzed in each cell by scRNA-seq
NCT05163106	Norway	Breast cancer	Using scRNA-seq to build a cellular atlas of whole tumor biomarkers for targeted cancer treatments
NCT05030805	France	Ovarian cancer	Identify the architecture and microenvironment of primary and secondary tumors of 50 patients with EOC by using scRNA-seq
NCT04816838	Republic of Korea	NSCLC (non-small cell lung cancer)	Investigating innate drug resistance and tumor microenvironment to osimertinib by performing scRNA-seq
NCT04658303	United States	Melanoma	The clonal evolution analysis of tumor cells through and pimonidazole-enabled scRNA-seq will be used to identify transcriptomic changes in tumor, immune, and stromal cells correlated with hypoxia exposure
NCT05677724	China	Primary liver cancer	Resolving the regulatory role of HBV on the hepatocellular carcinoma immune microenvironment by scRNA-seq
NCT05807516	Italy	Breast neoplasms	Study of the impact of neoadjuvant therapy on the heterogeneity of triple-negative breast cancer through scRNA-seq
NCT05398380	Spain	CRC (colorectal cancer)	Deep phenotyping of rare and common cell populations and determination of developmental trajectories of distinct cell lineages from the metastatic liver removed to the recurrence by using scRNA-seq
NCT05993858	China	HNSCC (head and neck squamous cell carcinoma)	Exploring the efficacy and safety of PD-1 inhibitor combined with cetuximab by scRNA-seq in neoadjuvant therapy for locally advanced HNSCC
NCT05397132	United States	Hematologic malignancy	Identify the correlation between change in molecular/genetic analysis and disease relapse/resistance in CAR-T therapy by scRNA-seq
NCT05807542	China	Esophagus cancer	Prediction for pCR after neoadjuvant immunotherapy combined with chemotherapy using scRNA-seq in patients with locally advanced esophageal squamous cell carcinoma (ESCC)
NCT06702891	Location not provided	Gastric cardia cancer	Exploring the heterogeneity, identifying specific biomarkers and potential therapeutic targets for cardia cancer by using scRNA-seq, and providing a basis for the development of new diagnostic tools and therapeutic strategies
NCT04888546	China	Hepatocellular carcinoma	scRNA-seq will be performed on the tissue samples punctured and the surgically resected specimens to explore the gene mutation sites related to efficacy
NCT05636605	China	Lung cancer	Analyzing tumor heterogeneity, mapping the microenvironment map of lung cancer and exploring the mechanism of sensitivity and resistance to anti-PD1/PD-L1 antibodies by using scRNA-seq
NCT04806334	United States	Bladder cancer	Patients with suspected bladder tumor will undergo novel 4D MRI imaging along with scRNA-seq in hopes of identifying a radiogenomic signature that can improve our staging of patients with muscle-invasive bladder cancer
NCT05304156	France	Acute myeloid leukemia	Apply scRNA-seq and multiparameter flow cytometry to correlate dynamic phenotypic landscapes with clinical outcomes
NCT05304858	United States	Prostate cancer	Profiling of the tumor microenvironment through scRNA-seq for deep profiling of the local immune microenvironment in the tumor
NCT04789252	Italy	Non-small cell lung cancer	Displayed high-resolution definition of myeloid cell subtypes and tumor-associated T cells, their phenotype, distribution within the tumor, as well as their functional characteristics by using scRNA-seq
NCT05807789	Italy	Hematologic malignancy	Study of CAR + and CAR- lymphoid populations and of myeloid populations with scRNA-seq in peripheral blood samples in order to understand the cellular dynamics related to clinical outcomes such as response to therapy and adverse events
NCT05789498	China	Esophageal cancer/lung cancer	This study aims to investigate the impact of immunotherapy on the immune status of tumor microenvironment and peripheral blood of chest cancer patients by scRNA-seq
NCT05676372	Belgium	CRC/PC (colorectal peritoneal carcinoma)	Comprehensively catalog the stromal cell types in the microenvironment of colorectal cancer peritoneal metastases utilizing scRNA-seq
NCT05558644	France	Thymic epithelial tumors	scRNA-seq was performed to assess the feasibility of characterizing the immune environment of TETs and the constitutional and somatic molecular profiles of patients with localized thymic epithelial tumor (TET)
NCT04352777	United States	Breast cancer	This study is to perform an in-depth analysis of changes in the tumor immune microenvironment in patients undergoing treatment with standard of care endocrine therapy and abemaciclib in the advanced setting via scRNA-seq
NCT04367025	China	Gastric cancer	Evaluating the efficacy and safety of perioperative chemotherapy plus PD-1 antibody by scRNA-seq in gastric cancer
NCT06489301	United States	Skin cancer	Determining the origin of cells called fibroblasts which are present after treatment with Fractionated Laser Resurfacing (FLR) by using scRNA-seq
NCT05878288	Australia	CSCC (cutaneous squamous cell carcinoma)	Comprehensively describe the molecular profile of the tumor ecosystem of cutaneous squamous cell carcinoma (CSCC) patients treated with neoadjuvant immunotherapy using scRNA-seq
NCT05894889	China	NSCLC (non-small cell lung cancer)	Evaluate the efficacy and safety of neoadjuvant pembrolizumab plus chemotherapy followed by pembrolizumab adjuvant in stage IIA-IIIB (N2) NSCLC participants without sensitizing e.g.FR/ALK mutation using scRNA-seq
NCT06476964	France	Skin cancer	Characterization of the immune cells infiltrate expression of multiple immune cell markers will be assessed in samples from different subtypes of cSCC by scRNA-seq
NCT06007989	United Kingdom	Myeloma	Using scRNA-seq to characterize the expression profile of myeloma cells in hypoxic areas of marrow
NCT04932343	Republic of Korea	NSCLC (non-small cell lung cancer)	Understand the metastasis in advanced NSCLC through comparing genomic and transcriptomic patterns between the circulating tumor cells and metastatic tumor cells by scRNA-seq
NCT06529731	United States	Acute myeloid leukemia	scRNA-seq conducted to understand the transcriptomic changes induced by IFN-γ in leukemia cell subsets, including those with stem cell characteristics
NCT06720454	China	PDAC (pancreatic ductal adenocarcinoma)	Reveal the gene regulatory network of pancreatic cancer by analyzing scRNA-seq and to draw a pancreatic cancer regulator map
NCT05675462	China	Hepatocellular carcinoma	Detection of systemic and local immune activation in tumors and peripheral blood mononuclear cell will be assessed by scRNA-seq
NCT06611072	Netherlands	Ovarian cancer	scRNA-seq will be performed to analyze cell composition and epigenetic status of cells of immune organs in ovarian cancer patients
NCT05157581	United States	CTCL (cutaneous T-cell lymphoma)	Evaluate immune responses post ECP using innovative technology such as scRNA-seq coupled with TCR sequencing to characterize ECP-related change in malignant cells
NCT04954339	Republic of Korea	Hepatocellular carcinoma	Distinct immunophenotypes and dynamic changes of tumor-infiltrating immune cells by scRNA-seq.
NCT03984578	Singapore	CRC (colorectal cancer)	scRNA-seq will be used to describe the enrichment of different immune cell states or cell types
NCT05048524	China	Pancreatic cancer	Biomarkers including but not limited to tumor mutation burden, change of immune cell proportion and percentage of Treg in the tumor microenvironment by scRNA-seq
NCT05723107	United States	Pancreatic cancer	Tumor biopsy will be taken for scRNA-seq during each endoscopic Ultrasound-Guided radiofrequency ablation (EUS-RFA) procedure
NCT05189782	China	Diffuse large B cell lymphoma	The abundance and phenotypes of immune cell subtypes within tumor or normal tonsil tissues measured by scRNA-seq
NCT05767684	China	Refractory tumor	Immune response biomarker study by scRNA-seq
NCT04656535	United States	Recurrent glioblastoma	Pharmacodynamic effects of each pre-surgery treatment will be evaluated with scRNA-seq of tumor and blood to determine effects of each intervention on the immune response
NCT04946773	United Kingdom	Cholangiocarcinoma/hepatocellular carcinoma	Proportion of immune cell populations relative to total immune cell count measured by scRNA-seq
NCT03558945	China	Pancreatic tumor	scRNA-seq will be performed to evaluate the percentage of the immune cell populations
NCT06239272	United States	Adipocytic neoplasm	scRNA-seq will be performed to evaluate the pretreatment, post-induction chemotherapy, and recurrent tumor material, and to characterize the longitudinal changes in tumor heterogeneity and tumor microenvironment
NCT06250010	Italy	Endometrial cancer	Spatial techniques will be exploited transcriptomics coupled to scRNA-seq to study interactions between cells in the TME and at the maternal-fetal interface
NCT06000787	United States	Glioma	Compared in participants with tumors showing high versus low tumor heterogeneity assessed quantitatively based on scRNA-seq
NCT06687876	Netherlands	Esophageal adenocarcinoma	scRNA-seq or SCENITH analysis will be exploited metformin to induce a metabolic switch in macrophages, T cells, and cancer cells
NCT04135079	Italy	Multiple myeloma	scRNA-seq will be exploited for a comprehensive analysis of immune transcriptomes, including but not limited to the gene expression profiles of high-resolution subsets of B cell, T cell, NK cell, and myeloid compartments
NCT06195150	Italy	Clear cell renal cell carcinoma	Comprehensively map intra and inter-tumor heterogeneity of ccRCC in VHL patients through the use of scRNA-seq
NCT04627246	Switzerland	Pancreatic adenocarcinoma	The assessment of the interactions between tumor cells and the immune system within the tumor microenvironment will be performed by scRNA-seq
NCT04622423	Italy	CRC (colorectal cancer)	scRNA-seq on sorted myelomonocytic and T cells infiltrating CRC/PDAC MTS samples
NCT05791149	France	Head and neck squamous cell carcinoma (HNSCC)	Construct a consolidated signature of four genes whose DNA is subject to methylation and gene expression is restricted to cancer cells, by crossing TCGA analysis with scRNA-seq
NCT05173298	Republic of Korea	Hepatocellular carcinoma	Precise classification and clinical significance of HCC based on scRNA-seq to discover biomarkers highly associated with treatment response in HCC patients who received atezolizumab and bevacizumab combination
NCT06014255	United States	Prostate cancer	Number of participants with scRNA-seq of tumor tissue in treated and untreated prostatectomies
NCT04638582	Canada	Lung cancer	scRNA-seq is defined as complete transcriptomic data from tumor epithelial, stromal, and immune compartments obtained from tumor samples
NCT03743766	United States	Melanoma	scRNA-seq will be performed to evaluate the presence and quantity of RNA in blood and tumor tissue
NCT06626893	Italy	Leukemia	scRNA-seq will be performed to improve the understanding of the genetic and molecular alterations of disease
NCT06208735	Canada	B-cell leukemia	Clonal typing and gene expression profiling of the CLIC-2201 infusion product by scRNA-seq
NCT06049381	China	Relapsed non-Hodgkin lymphoma	Changes of characteristics of tumor microenvironment will be analyzed between pre-treatment and post-treatment biopsy samples of patients by scRNA-seq
NCT05281003	China	Esophageal squamous cell carcinoma	Single-cell genomic measurement of hypoxia pathway genes is assessed by scRNA-seq
NCT04495894	United States	NSCLC (non-small cell lung cancer)	scRNA-seq will be performed to evaluate the effects of ketorolac on immune response pathways
NCT06200974	Canada	Prostate carcinoma	scRNA-seq of immune cell populations before and after 13.5 gray of radiation will be used for each individual patient and the number of patients with a change in gene expression
NCT03635164	United States	HNSCC (head and neck squamous cell carcinoma)	Analysis of gene expression of RNA levels and entire genome sequences using the 10X Genomics scRNA-seq platform
NCT06109233	China	Multiple myeloma	scRNA-seq was applied to analyze and compare the differences in MM cells (clones) and bone marrow microenvironment (including immunity, inflammation, and stroma) of MRD-transformed and non-transformed patients after adjusting the treatment regimen
NCT05923879	China	Lymphoma	Tumor tissues collected before treatment and at the point of progression available for scRNA-seq after quality control
NCT03869034	China	Hepatocellular carcinoma	Using scRNA-seq to find biomarkers of treatment response by investigating the variation of tumor and immune cells before and after treatment

Biomaterials are a large catalog of materials ranging from organic components to bio-inert inorganic and metallic implants developed for various biomedical applications. In the field of cancer immunotherapy, apart from being employed as tumor-targeted or locoregional drug delivery vehicles to improve the pharmaceutical and safety profiles of therapeutics, the intrinsic properties of these rationally tailored biomaterials could allow direct reprogramming of the TME to markedly reinforce the therapeutic potency of cancer immunotherapies [16]. Therefore, utilizing scRNA-seq to comprehensively understand the impacts of biomaterials on the TME is recognized as an efficient strategy to screen effective biomaterials for enhanced cancer immunotherapy.

In the review article, we would first introduce the history of scRNA-seq, followed by introducing their current utilization status in the field of cancer immunotherapy and biomaterials for precise predicting the prognosis of cancer immunotherapy and fast identification of TME modulating biomaterials for enhanced cancer immunotherapy.

## Overview of scRNA-seq

**Figure 1:**
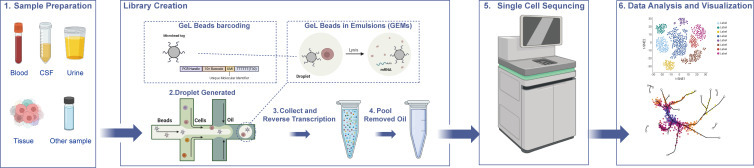
Workflow of single-cell RNA sequencing The types of samples include blood, cerebrospinal fluid (CSF), urine, various tissue samples, and other sample types. 2. In the microfluidics system for cell sorting, gel beads with barcodes are introduced from the left at a constant speed, while the cells and enzymes to be sorted are introduced from the bottom at specific time intervals. These components combine with the gel beads and subsequently enter the oil phase to form Gel Bead-in-Emulsions (GEMs). The barcode on each gel bead carries a Unique Molecular Identifier (UMI) specific to each cell. During the subsequent library construction, any complementary DNA (cDNA) amplified from this cell will incorporate the unique molecular identifier (UMI) along with the preceding 10 × barcode. Consequently, cDNA amplified from distinct cells can be differentiated. Following the generation of Gel GEMs, the cells undergo lysis and reverse transcription (RT) to synthesize cDNA, culminating in the formation of a cDNA library. These library construction processes are confined within GEMs, which function as a form of physical isolation, ensuring that each individual cell is contained within a distinct ‘chamber’. Furthermore, given that GEMs create an emulsion within the oil phase, tens of thousands of microdroplets are generated, with each microdroplet representing an individual GEM. This configuration facilitates a high-throughput approach to single-cell RNA sequencing. Subsequently, the GEMs were disrupted using a degreasing reagent, and the cDNA was purified using magnetic beads before being amplified via PCR to produce a stable cDNA library. Following the construction of the cDNA library, sequencing was conducted utilizing a high-throughput sequencing platform. Initially, employ Cell Ranger to extract the cell’s barcode and Unique Molecular Identifier (UMI), align the sequences to the reference genome, and calibrate the barcode. Proceed to filter and calibrate the UMI, followed by its quantification. Utilize an algorithm to differentiate between barcodes associated with actual cells and those representing background noise, thereby isolating genuine single-cell data. This process culminates in the generation of a gene expression matrix for each cell. Subsequently, apply Seurat for additional cell filtering and standardization and clustering (K-Means), visualization (t-SNE) and UMAP dimensionality reduction are performed through dimensionality reduction (PCA) to obtain common cell clustering typing results, marker genes and differentially expressed gene information.

The advent of scRNA-seq technology was initially documented by Tang et al. in 2009. In 2011, Islam et al. pioneered the establishment of the first multiplexed scRNA-seq library, providing a foundational framework for the burgeoning popularity of scRNA-seq. Currently, numerous scRNA-seq platforms, including 10 x Genomics, BD Rhapsody, Fluidigm C1, Singleron Matrix, and BGI DNBelab C4, are the most popular. 10 x Genomics and BGI DNBelab C4 have been developed and applied for distinct scenarios in clinic. For instance, 10 x Genomics and BGI DNBelab C4 are cost-effective for large-scale research, BD Rhapsody and Singleron Matrix excel in multi-omics compatibility for complex samples, while Fluidigm C1 is ideal for low-throughput, high-precision needs, such as rare samples or high-depth analysis. The detailed comparison of these mainstream platforms is summarized and shown in Table 2. The fundamental principle underlying scRNA-seq involves the extraction of RNA from individual cells, followed by the synthesis of complementary DNA (cDNA) via reverse transcription and subsequent high-throughput sequencing. Therefore, the typical workflow encompasses several key steps, including the dissociation of tumor tissue into single cells, the isolation of individual cells, RNA extraction, cDNA synthesis, library construction, and sequencing (Figure 1) [17–19]. With the fast advancements in sequencing technology, scRNA-seq has achieved significant progress, particularly in sequencing depth, throughput, and cost-effectiveness. Furthermore, it can be integrated with other technologies, such as T-cell receptor (TCR) and B-cell receptor (BCR) sequencing, genome, epigenome, immunogenome, proteome, and so on, to offer more comprehensive cellular feature information [17,18]. These technological advancements have significantly benefited the extensive application of scRNA-seq in various biomedical domains including oncology, immunology, and developmental biology [17,19].

**Table 2:** Comparisons of mainstream platforms of scRNA-seq

Platform	Technical principle	Detection of single cell number	Cell capture efficiency	Sequencing depth	UMI calibration method	Application scenario
10 x genomics	Based on microfluidic GEM technology, single cells and gel microbeads are encapsulated by oil-in-water droplets, and single-cell labeling is achieved by combining Barcode and UMI	1,000–10,000 cells per channel, 500–80,000 cells total throughput per 8 channels	High (polycystic rate < 0.9%)	Medium (50,000–100,000 reads/cell)	UMI deduplication and quality filtering based on molecular tag counts by cell ranger	Cancer heterogeneity analysis, immune cell atlas, developmental biology, multi-omics research
BD rhapsody	Based on micropore sedimentation technology, an array of more than 200,000 micropores captures single cells through natural sedimentation, combined with an imaging system for real-time quality control	Theoretically, tens of thousands of cells can be processed simultaneously	High (polycystic rate < 1%)	Medium (50,000–200,000 reads/cell)	Based on UMI molecular counting, SeqGeq software has a built-in correction algorithm to support batch effect removal	Multi-dimensional analysis of complex diseases, drug target screening, and cross-species sample research
Fluidigm C1	Capture single cells based on microfluidic chips and automate lysis, reverse transcription, and pre-amplification	96 or 800 single cells per chip, low throughput but high precision	High (>90% capture of rare cells)	High (200,000–500,000 reads/cell)	No UMI design, relying on amplification cycle number normalization and biological replicate validation	Rare cell research, drug stimulation experiments, single-cell methylation analysis, early development research
Singleron matrix	Based on high-precision microfluidic chips combined with Poisson distribution principle, single cells are separated automatically and nucleic acids are captured by magnetic beads	High-density chip can capture 30,000 cells on a single chip	High (polycystic rate < 2%)	Medium (100,000–200,000 reads/cell)	Based on UMI molecular tags, CeleScope software uses clustering deduplication and sequencing error correction algorithms	Immune repertoire analysis, frozen tissue research, cross-species multi-sample mixed testing
BGI DNBelab C4	Based on microfluidic droplet generation technology, dual magnetic bead system (mRNA capture magnetic beads + droplet recognition microbeads)	Efficiently capture 20,000 cells in a single experiment	High (polycystic rate < 1%)	Medium (50,000–150,000 reads/cell)	Dual magnetic beads synergistic correction, UMI counting combined with droplet identification code double filtering to reduce false positives	Public health surveillance, medical research, spatial transcriptome integration, large-scale cohort studies

### scRNA-seq facilitates the prediction of immunotherapeutic efficacy

Tumor-infiltrating immune cells including T cells, B cells, and myeloid cells are important components of the TME of solid tumors, and their intratumorally abundance and activation status play a pivotal role in determining the response of solid tumors to cancer immunotherapy and other treatments by impairing the priming of adaptive immune responses[20] [21]. Therefore, comprehensive understanding of the composition and functionality of immune cells within the TME has recently proven to be a valuable strategy to predict the prognosis of immunotherapeutic interventions[22]. Attributing to its superiority in the high-throughput identification of intratumoral immune cell subsets and their characteristic markers, preoperational scRNA-seq has been intensively explored to screen out the ‘right patients’ who would be sensitive to certain cancer immunotherapies (Table 3).

**Table 3:** The predictive ability of scRNA-seq for the efficacy of tumor immunotherapy

Cell type	CD8 + T **cells**	CD4 + **T**	Myeloid cells	B cells	CAR-T cells
The predictive ability of scRNA-seq for the efficacy of tumor immunotherapy	scRNA-seq can reveal the association between CD8^+^T cell characteristics and ICB efficacy; the proportion of specific CD8^+^T cell subsets can predict the benefit of anti-PD-1 therapy; the IL-21-driven pathway in different immunotherapies causes CD8^+^PD-1^+^T cells to present different transcriptional profiles and differentiation pathways.	scRNA-seq revealed that CD4^+^T cells have diverse functions, which are significantly associated with ICB therapy and have a positive impact on ICB prognosis; combination therapy can increase Th1-like CD4^+^ effector T cells. It was clarified that the 4PD-1hi CD4^+^ subpopulation is associated with high tumor mutation load.	scRNA-seq analysis of pan-cancer myeloid cells revealed that LAMP3 + dendritic cells are widely present; macrophages with high expression of CD73 in glioblastoma are associated with immunosuppression and poor anti-PD-1 efficacy; high expression of IL-8 in myeloid cells is associated with poor ICB efficacy; high scores of specific macrophage clusters in breast cancer are associated with good prognosis, providing targets and markers for immunotherapy.	scRNA-seq can predict the prognosis of ICB therapy by analyzing the gene expression characteristics of TIBs: in neoadjuvant ICB treatment of melanoma, responders have higher CD20 + B cell density, tertiary lymphoid structures (TLS) and TLS to tumor area ratio; scRNA-seq can identify B cell-related genes and other immune cell genes in TLS-rich melanoma for prognosis prediction; TCF7 + naive/memory T cells increase in B cell-enriched tumors, suggesting that B cell-rich TLS are critical to shaping the immune microenvironment.	scRNA-seq can analyze the transcriptional characteristics of CAR-T cells to predict their functions: in CD19-CAR-T therapy, specific CD8^+^T cells and exhaustion markers before infusion are associated with treatment failure; in patients with large B-cell lymphoma, some/non-responders have defective CAR-T killing function due to abnormal tumor cell pathways.

The antitumor efficacy of ICB therapeutics is contingent upon the extent of tumor-infiltrating CD8^+^ T cells recognizing and eliminating tumor cells [23]. The precise identification of the abundance and activation status of CD8^+^ T cell subsets in tumors is recently recognized as an indicator for assessing the effectiveness of immunotherapy. An scRNA-seq analysis of 16,291 immune cells isolated from 48 melanoma patients treated with ICB immunotherapy showed that the tumor-infiltrating CD8^+^ T cells of these patients exhibited distinctive phenotypes. It was discovered that the clinical responses to ICB in patients with melanoma were associated with the presence of stem-like CD8^+^ T cells with reduced expression of co-inhibitory molecules and elevated memory, activation, and cell survival transcriptional and epigenetic programs compared with exhausted CD8^+^ T cells [24,25]. In another study, scRNA-seq of 33 melanoma tumors together with in-depth computational analyses identified that a resistance program of malignant cells is tightly associated with T cell exclusion and immune evasion [23]. A single-cell cohort study demonstrated that the patients with stage IV melanoma with a higher baseline ratio of circulating Ki67^+^PD-1^+^CD8^+^ T cells in peripheral blood relative to tumor burden would benefit from pembrolizumab treatment demonstrated [26]. Comparable results have also been observed in patients with non-small cell lung cancer (NSCLC) undergoing anti-PD-1 therapy [27]. A recent study found that the combinational use of scRNA-seq and TCR sequencing revealed that IL-21-driven STAT1 or STAT3 pathways create different transcriptomic profiles in CD8^+^PD-1^+^ T cells during the combined anti-CTLA-4 and anti-PD-1 therapies compared with anti-PD-1 therapy alone. It was also uncovered that IL-21 signaling was more pronounced in anti-CTLA-4 therapy than in anti-PD-1 therapy, indicating that PD-1^+^CD8^+^ T cells follow different differentiation paths in various immunotherapies [28].

CD4^+^ T cells were traditionally regarded as auxiliary cells primarily responsible for activating CD8^+^ effector T cells. However, advancements in technologies such as scRNA-Seq have revealed that CD4^+^ T cells perform diverse roles within the TME. Notably, accumulating evidence indicates that CD4^+^ T cells possess independent functions that contribute to the priming of anti-tumor immunity [29]. A comprehensive understanding and investigation of the functions and regulatory mechanisms of CD4^+^ T cells are therefore crucial for the advancement of novel tumor immunotherapies and the enhancement of treatment efficacy. Recent investigations have indicated that CD4^+^ T cells positively influence the prognosis of ICB therapy [30]. For instance, tumor models responsive to CTLA-4 blockade commonly have a higher CD4^+^ T cell population [31], and the presence of pre-existing CD4^+^ T cells in the bloodstream has been shown to be associated with improved clinical outcomes in melanoma patients undergoing anti-CTLA-4 treatment [32]. Additional research has demonstrated that the combination of anti-CTLA-4 and anti-PD-1 therapies can augment the proportion of Th1-like CD4^+^ effector T cells [33]. In addition, the melanoma patients with combined anti-PD-1 and anti-CTLA-4 therapies revealed PD-1-expressing CD4^+^FOXP3^-^ T cells (4PD-1^hi^), a unique suppressive T cell subset linked to higher tumor burden. scRNA-seq shows that the presence of 4PD-1^hi^ cells predicts poor treatment outcomes [34]. Considering different T cell subsets constitute critical elements of the TME, thorough and nuanced analyses of the distinct roles and regulatory mechanisms of T cell subsets are essential for advancing the development of more effective tumor immunotherapeutics and selecting reasonable and effective treatments for each patient.

While T cells have been the primary focus of ICB therapies, tumor-infiltrating myeloid cells (TIMs), which are both abundant and diverse within the TME, have not been as extensively studied. Although the research on TIMs is expanding, their influence on the prognosis of ICB therapies remains incompletely understood. Advances in scRNA-seq have significantly improved our capacity to examine and interpret the heterogeneity and intercellular interactions among various TIM subtypes within the TME [35–38]. A recent study conducted a comprehensive pan-cancer scRNA-seq analysis of myeloid cells, utilizing 192 samples from eight distinct cancer types, with the objective of elucidating the spectrum of TIM phenotypes and functionalities in both treatment and post-treatment patients [39]. Another pan-cancer scRNA-seq analysis demonstrated that LAMP3^+^ conventional dendritic cells (cDCs) are widely present, extending the conclusion of their diverse origins across all cancer types [39]. scRNA-seq also has been employed to identify a distinct subset of macrophages in glioblastoma multiforme, characterized by elevated expression of CD73, and which is found to contribute to immunosuppression in glioblastoma and reduced overall survival in patients with anti-PD-1 therapy [40]. Numerous studies have previously documented a correlation between increased plasma IL-8 levels and negative outcomes following ICB therapy [41]. In patients with metastatic urothelial carcinoma and metastatic renal cell carcinoma undergoing Atezolizumab treatment, scRNAseq of the immune compartment showed that IL-8 is primarily expressed in circulating and intratumoral myeloid cells and the high IL-8 expression is associated with down-regulation of the antigen presentation machinery. Consequently, mitigating the IL-8-mediated inflammatory response in myeloid cells may enhance the prognosis of patients undergoing ICB therapy [42]. An integrated analysis of scRNA-seq data of breast cancer identified five issue-resident macrophages (RTM) clusters with mixed M1-M2 macrophage phenotypes. High RTM cluster scores are shown to be correlated with increased CD8^+^ T cells, M1 macrophages, dendritic cells, and better survival in responders [43]. These studies demonstrated that progressively refining the characterization of myeloid cells in TME through scRNA-seq is a promising strategy to identify more valuable therapeutic targets and predictive markers for effective immunotherapy.

B cells, as a pivotal component of the immune system, play a crucial role in immunity [44,45]. However, they have received comparatively less attention in the field of cancer immunotherapy, and the compositional and functional heterogeneity of tumor-infiltrating B cells (TIBs) has not been systematically investigated [46]. Preclinical studies have demonstrated that neoadjuvant ICB therapy enhances OS rates and antigen-specific T cell responses in a murine spontaneous metastatic breast tumor model compared with adjuvant therapy [47]. In a randomized phase II clinical trial (NCT02519322), high-risk resectable melanoma with neoadjuvant ICB treatment (nivolumab plus ipilimumab) exhibited a high therapeutic efficacy of 45%, representing an 80% increase over the 25% of the patients with nivolumab monotherapy. Meanwhile, it was uncovered that the patients responding to the neoadjuvant ICB treatment exhibited a higher density of CD20^+^ B cells and tertiary lymphoid structures (TLS), as well as an increased ratio of TLS to tumor area, compared with non-responders. Accumulating evidence reveals that scRNA-seq could be employed to identify the gene expression features of TLS-rich melanomas, including the B cell-specific expression gene CD79B, the B cell activation indicative gene CCR6, and genes associated with other immune cell types, for predicting the prognosis of patients with ICB therapy. It has also been uncovered that tumors enriched with B cells have an increased presence of TCF7^+^ naive T cells and/or memory T cells, suggesting that B cell-rich TLSs play a critical role in shaping the immune microenvironment of melanoma [48]. These studies highlight the practical value of utilizing sc-RNA-seq to analyze B cell gene expression features in predicting the prognosis of ICB immunotherapy.

CAR-T cells are ‘personalized’ therapeutic products generated from the patient’s own T cells but often exhibit inconsistent quality and significant variability in clinical practices, thereby making the therapeutic efficacy of CAR-T cell therapy hard to predict. A Phase I clinical trial investigating CD19-CAR-T cells for relapsed/refractory CD19^+^ leukemia revealed that an elevated frequency of LAG-3^+^/TNF-α^low^CD8^+^ T cells in peripheral blood prior to the cell infusion, along with the expression of exhaustion markers, was correlated with ultimate treatment failure [49,50]. scRNA-seq was employed to analyze autologous axicabtagene ciloleucel (axi-cel) CD19-CAR-T cell infusion products, with the aim of identifying their transcriptomic features linked to efficacy and toxicity in a cohort of 24 patients diagnosed with large B cell lymphomas (LBCLs). The study demonstrated that the patients who achieved a complete response, as determined by positron emission tomography/computed tomography (PET/CT) at their three-month follow-up, exhibited a three-fold increase in the frequency of CD8^+^ T cells expressing memory signatures compared with those with partial response or progressive disease. This finding indicates that distinct T cell subsets are associated with varying clinical outcomes [51]. Further analysis of scRNA-seq results of CAR-T cells derived from the LBCL patients exhibiting partial or non-response revealed that the compromised death receptor signaling pathway in tumor cells resulted in the failure of the cytotoxic killing function of CD19-CAR-T cells. This dysfunction of CD19-CAR-T cells indicates a novel mechanism of resistance to CAR-T cell therapy that operates independently of antigen presence [52]. These results highlight that scRNA-seq is a valuable tool to elucidate the transcriptomic characteristics of CAR-T cells throughout treatment for identifying predictive biomarkers for achieving personalized immunotherapy.

Despite the transformative advancements of ICB therapy in clinical, its mechanism of action primarily reversing immune cell suppression rather than directly targeting tumor cell intrinsic pathways necessitates further investigation into the TME and the interactions between tumor and immune cells. This research is crucial for elucidating the determinants of ICB response. To identify the correlation of tumors and their immune characteristics, researchers have developed multifaceted biomarker prediction models by integrating various methodologies, including scRNA-seq, spatial imaging, and multi-omics approaches. These integrative models have substantially enhanced the predictive accuracy of ICB treatment outcomes. Moreover, the assessment of tumor intrinsic properties and tumor immune signals has demonstrated superior predictive capabilities. For instance, comprehensive batch sequencing techniques that concurrently evaluate tumor and immune characteristics outperform analyses based on single variables. These findings underscore the importance of adopting a holistic and integrated approach to modeling the therapeutic effects of ICB [53]. With the help of expanding single-cell data and machine learning, predicting pan-cancer single-cell immunotherapy models is now feasible. By categorizing single-cell transcriptional profiles of various cancers and cell types into ecosystem subtypes, researchers identified a strong correlation between a specific subtype and ICB response, supported by five ICB scRNA-Seq cohorts. This immunotherapy-responsive ecotype was conserved in at least 11,001 tumor samples across 32 cancer types. A comprehensive analysis identified significantly overexpressed genes linked to this ecotype, forming a pan-cancer immunotherapy-responsive signature called immunotherapy-responsive ecotype signature [54].

Another study aimed to identify tumor heterogeneity among patients with the same disease and within different cells of a single patient. It introduced comboSC, a computational tool for optimizing tumor therapy using personalized scRNA-seq data. ComboSC assesses the tumor’s immune microenvironment using the Tres model’s immune score to match suitable immunotherapy or targeted therapy. For tumors unsuitable for direct immunotherapy, it employs a bipartite graph model to optimize combinatorial therapy. Specifically, for low immune score tumors, it optimizes drug combinations for complex cell groups. For tumors with medium immune scores, the approach employs an immune recovery strategy alongside bipartite graph modeling of selected immune-related trajectories. This method considers current RNA expression and projected RNA velocity trends to identify small molecules that can be paired with ICB to enhance immunotherapy. Ultimately, comboSC offers therapy combinations ranked according to their response scores. The entire comboSC pipeline can be readily applied to customized datasets via the comboSC online web server [55].

Apart from providing descriptive profiling of the TME, scRNA-seq offers additional technical supports to further promote the precision treatment of cancers. A series of reports have demonstrated that scRNA-seq could also be utilized to benefit biomarker identification, therapeutic efficacy stratification, and treatment decision-making. For example, 1) scRNA-seq enhances tumor biomarker identification by moving from ‘pan-tumor’ to ‘subpopulation specificity’. Unlike traditional bulk sequencing, which might miss cell heterogeneity, scRNA-seq identifies markers at a single-cell level, improving sensitivity and clinical applicability by pinpointing specific tumor cell subpopulations, immune cell functions, or microenvironment traits [56]. 2) It also enhances tumor efficacy stratification by shifting from ‘empirical grouping’ to ‘molecular typing’: It accurately categorizes patients into ‘potential benefit’ and ‘drug resistance high-risk’ groups, minimizing side effects and resource waste from ineffective treatments. By analyzing single-cell heterogeneity before and during treatment, scRNA-seq addresses the issue of varying efficacy at the same stage seen in traditional methods like TNM staging [56,57]. 3) scRNA-seq also aids in tumor treatment decisions by enabling ‘individualized dynamic adaptation’. It tackles the challenge of selecting and adjusting the best treatment plan for each patient by providing insights into cellular mechanisms of treatment response, such as the clearance of sensitive subpopulations, expansion of resistant ones, and immune microenvironment changes [58].

scRNA-seq breaks down tumor cellular heterogeneity, shifting treatment from population-based to precision medicine at a single-cell level. It identifies specific biomarkers, enables detailed efficacy stratification, and guides treatment decisions, including regimen selection and drug resistance strategies. As costs decrease and spatial transcriptome technology integrates, scRNA-seq will enhance multidimensional decision-making, advancing tumor treatment toward individualized dynamic adaptation. In conclusion, a diverse array of models for predicting ICB responses are presently undergoing trials, potentially enhancing the accuracy and efficacy of immunotherapy. While individual patient-level predictive analysis is optimal, it is likely to serve as a component within a broader multianalyte algorithm. The most effective multimodal biomarker models will offer a longitudinal evaluation of efficacy of immunotherapy by incorporating factors related to the tumor, immune microenvironment, and host.

## Biomaterials modulate the TME to enhance the efficacy of immunotherapy

Immunotherapy, as a powerful strategy for cancer treatment, has achieved tremendous efficacy in clinical trials. Despite these advancements, there is much to do in terms of enhancing therapeutic benefits and decreasing the side effects of immunotherapy. Advanced biomaterials and nanomedicine, including liposomes, polymers, and silica, play a vital role in the codelivery of drugs and immunomodulators. To date, diverse biomaterial strategies have been demonstrated to be able to attenuate tumor hypoxia, neutralize tumor acidity, eliminate intratumoral reactive oxygen species, or decrease intratumoral interstitial pressure, thereby markedly reverse tumor immunosuppression to benefit cancer immunotherapy and many others. These pioneering studies indicated the comprehensive understanding of the modulating mechanisms of these biomaterials on TME would facilitate the screening of effective ones with translational possibility, aiming at providing more tools for enhanced cancer immunotherapy [59–61].

Evidence shows that the physiochemical properties of biomaterials variably affect immune cell states, complicating the profiling of the TME, which is crucial for treatment decisions and response assessment. For instance, biomaterial surface morphology and topology significantly influence macrophage phenotype polarization. MXene/hydroxyapatite coatings with micro-nano wrinkles activate the FAK/Src pathway through integrin-mediated signals, promoting macrophage polarization to the anti-inflammatory M2 phenotype and enhancing osteoblast differentiation. This study underscores the synergistic role of topological structures and chemical signals in regulating the bone immune microenvironment [62]. The micro-nanotube design on titanium surfaces promotes macrophage M2 polarization through the ERK-Beclin-1-autophagy pathway. Blocking autophagic flux significantly lowers the anti-inflammatory marker Arg-1, indicating that surface topology affects immune response by altering autophagy. Likewise, the microgrooves and microcolumns in GelMA hydrogels adjust cytokine release, such as TNF-α and IL-10, by changing macrophage spreading and tension, showing that surface shape can directly influence inflammation levels [63,64]. Substrate stiffness affects B cell adhesion and activation through the PKCβ-FAK pathway, with stiff substrates (>10 kPa) boosting FAK phosphorylation and BCR signaling, while soft substrates (<1 kPa) hinder activation, influencing the adaptive immune response [65]. Modifying collagen hydrogel viscoelasticity can steer T cell differentiation into effector (high elasticity) or memory (high viscosity) subsets via the AP-1 pathway, offering a new method for enhancing adoptive cell therapy [66]. Introducing negatively charged amino acids like Asp on antigens improves electrostatic adsorption to aluminum adjuvants, prolongs antigen retention, and exposes neutralizing epitopes, enhancing immune responses [67]. Hydrophilic titanium surfaces selectively adsorb fibronectin and fibrinogen, activating the integrin β1/β2-PI3K/NF-κB pathway, polarizing macrophages to the M2 phenotype, which aids anti-inflammatory implant design [68]. Degradation of poly(β-amino ester) (PBAE) nanoparticles influences dendritic cell activation: high molecular weight enhances maturation via TLR4, while low molecular weight suppresses immune responses, emphasizing the role of degradation kinetics in vaccine carriers [69]. Chiral peptide hydrogels modulate IL-10 and T cell exhaustion, with type D gels enhancing immunosuppression and type L gels promoting anti-tumor responses [70]. Mucin coatings’ immunomodulatory effects depend on substrate mechanics: they maintain an anti-inflammatory phenotype on soft polyacrylamide but induce pro-inflammatory responses on hard polystyrene, highlighting the importance of chemical-mechanical synergy in immunomodulation [71]. These studies demonstrate that biomaterial properties—such as morphology, mechanics, chemistry, and degradation—affect immune cell activity from molecular mechanisms to practical applications. Surface topology affects the cytoskeleton through mechanical signals, stiffness influences signaling pathways, surface charge and degradation products alter the transcriptome, and viscoelasticity and degradation kinetics shape the immune microenvironment. These findings provide a basis for creating advanced immunomodulatory materials, including anti-inflammatory implants, vaccine carriers, and tumor immunotherapy platforms.

**Figure 2:**
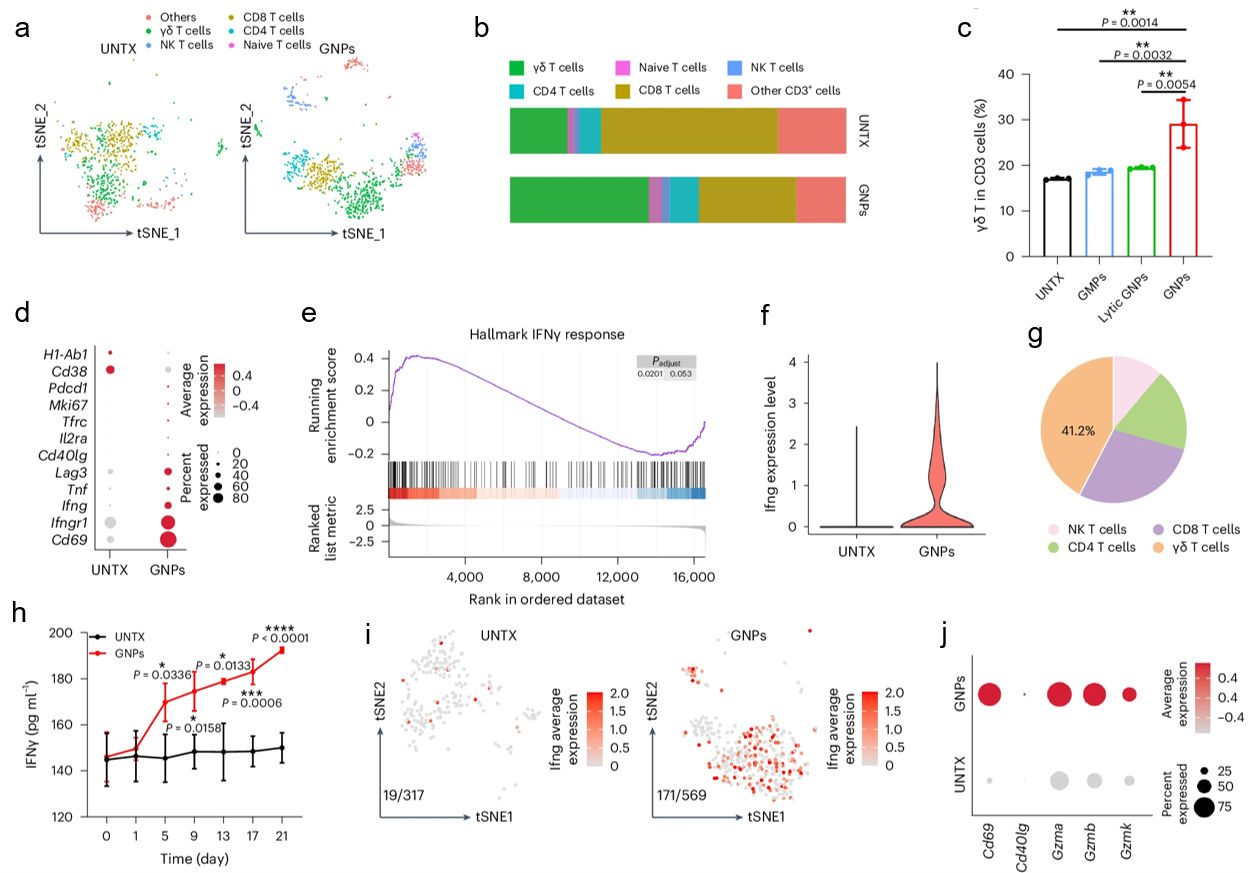
GNPs induced intestinal IFNγ-producing γδ T cells. (**a**) t-SNE visualization depicting different T cells clusters in the UNTX and GNP groups. (**b**) Proportional distribution of T cell subsets: γδ T cells, naive T cells, NK T cells, CD4 + T cells and CD8 + T cells. (**c**) Quantification of γδ T cells frequency within CD3 + T cells in four groups. (**D**) Expression profiles of activation markers and cytokine production by γδ T cells in UNTX and GNP groups. (**e**) Key signaling pathways associated with intestinal γδ T cells. (**f**) IFN-γ expression levels in T cells from UNTX and GNP groups. (**g**) Relative contributions of intestinal T cell subsets to IFN-γ production. (**h**) Temporal dynamics of circulating IFN-γ levels in UNTX- and GNP-treated mice. (**i**) T-SNE mapping of IFN-γ-γδ T cells within intestinal tissues of UNTX and GNP groups. (**j**) Dot plot of the activation markers of IFNγ-γδ T cells in the intestine of the UNTX and GNP groups. The figures was adapted from reference [74], and approved for reuse by Springer Nature. GNPs, garlic-derived nanoparticles; IFN-γ, interferon-γ

In the past several years, growing evidence suggests that scRNA-seq is a powerful tool to comprehensively assess the interactions between biomaterials and TME [72]. It is shown to be able to facilitate the elucidation of the impacts of biomaterials on specific cell subpopulations within the TME, as well as the understanding of how these modifications influence tumor progression and therapeutic response. In 2020, My Kieu Ha and her colleague employed scRNA-seq to analyze the diverse interactions between silver nanoparticles (AgNPs) and primary immune cells. It was demonstrated that polyethylenimine-coated AgNPs with positively charged surface exhibited a greater association with monocytes and B cells compared with other cell subsets. scRNA-seq data revealed that the two cell types exhibited differential responses to AgNPs treatment. Specifically, B cells demonstrated NRF2-mediated oxidative stress, whereas monocytes engaged in Fcγ-mediated phagocytosis [73]. In addition, scRNA-seq was also utilized to demonstrate that garlic-derived nanoparticles (GNPs) significantly enhanced the proliferation, activation, and recruitment of endogenous γδ T cells within the intestine, resulting in the production of substantial amounts of IFN-γ (Figure 2). The translocation of γδ T cells and IFNγ from the intestine to extraintestinal subcutaneous tumors altered the tumor immune microenvironment and synergized with anti-PD-L1 therapy to induce a robust anti-tumor immunity [74].

Magnetic nanoparticles (MNPs), owing to their inherent magnetic properties, can be activated either by alternating magnetic field heating or by non-heating low-frequency magnetic fields. This activation can result in intrinsic therapeutic effects or facilitate drug release. Furthermore, the localization of MNPs at designated sites can be effectively tracked using magnetic resonance imaging (MRI) or magnetic particle imaging (MPI). The responsiveness of MNPs to external magnetic fields facilitates their remote manipulation, enabling the development of advanced nanosystems to modulate TME for enhanced cancer treatment. In a recent study, MNPs functionalized with pH low insertion peptides (pHLIP) were utilized to create a sophisticated pH-responsive tumor-targeted drug delivery system. Within the acidic TME, pHLIP can adopt an alpha-helical conformation, allowing it to insert into and traverse the tumor cell membrane, thereby serving as an intelligent tumor-targeting ligand. As a result, researchers discovered that such MNPs could infiltrate tumor tissue via vascular rupture and endothelial cell endocytosis. Subsequent scRNA-seq was employed to identify the cell populations that internalized MNPs within the tumor tissue, revealing a preferential accumulation of MNPs in regulatory Trem2^+^ TAMs [75]. These results highlight that scRNA-seq could be utilized to investigate the interactions between nanoparticles and specific cells in the TME.

In addition, scRNA-seq was employed to assess the efficacy of a nanoparticle formulation of the CDK4/6 inhibitor palbociclib (POx-Palbo). The results indicated that the combination of POx-Palbo with Sapanisertib holds potential as an effective therapeutic candidate for SHH medulloblastoma [76]. Furthermore, scRNA-seq analysis revealed that IgG1 reduced immune inhibitory signaling, enhanced MHC signaling from B cells to CD8^+^ T cells, and enriched the profiles of anti-tumor T cell and B cell receptors. The E285K-mAbs were additionally generated in the dimeric IgA (dIgA) format, with their anti-tumor efficacy contingent upon the polymeric immunoglobulin receptor (PIGR), a membrane-bound immunoglobulin receptor. In contrast, the anti-tumor activity of IgG1 was dependent on TRIM21, an intracellular immunoglobulin G receptor [77].

Through the analysis of alterations in the immune microenvironment following biomaterial treatment via scRNA-seq, researchers can attain a more profound comprehension of the dynamic responses of immune cells and their reactions to treatment. This approach serves as a potent tool for assessing the capacity of biomaterials to enhance different tumor treatment strategies. Looking forward, as scRNA-seq technology advances, the application of biomaterials in tumor immunotherapy is anticipated to become increasingly precise and personalized, offering novel insights into overcoming tumor immune evasion and drug resistance.

## Future directions and challenges

The rapid advancement of scRNA-seq has introduced new opportunities for clinical translation. This technology enables the detailed analysis of gene expression variability at the single-cell level, providing essential insights into the study of complex diseases, such as cancer and immune disorders [7]. In terms of clinical applications, scRNA-seq aids in the identification of specific cell types within the TME, thereby informing the development of personalized treatment strategies. An example of such an application is the radiotherapy-activated nanomaterial NBTXR3. Composed of hafnium oxide nanoparticles, NBTXR3 enhances cancer cell destruction and tumor control, with multiple clinical trials (NCT02379845, NANORAY-312) demonstrating its immunomodulatory effects. It enhances the efficacy of proton beam therapy and anti-PD-1 antibody therapy by increasing CD8^+^ T cell infiltration and up-regulating cytotoxic genes such as GZMB and IFNG. Furthermore, it promotes dendritic cell maturation, stimulates the secretion of proinflammatory cytokines, and inhibits the immunosuppressive functions of M2 macrophages [78–81]. Nonetheless, despite its promising potential, scRNA-seq technology continues to encounter numerous challenges in the clinical implementation process [82].

First, the inherent complexity and substantial costs associated with the scRNA-seq constrain its widespread adoption in routine clinical settings [83]. Although the current cost of scRNA-seq is relatively high, the development and application in technologies, methods, strategies, and reagents for all aspects of scRNA-seq will reduce costs as these methods are integrated into the clinic. Additionally, data analysis presents a significant hurdle. Extracting meaningful insights from extensive single-cell datasets, integrating data from diverse sources, and employing advanced computational methods for analysis and interpretation necessitate the development of more sophisticated algorithms and tools [84,85]. In order to achieve the reproducibility of results, it must have a standard single-cell acquisition process. In addition, standard reference data of cell subpopulations needs to be established to ensure the stability of cell classification results.

Furthermore, technical challenges exist in the acquisition and processing of clinical samples. Ensuring the integrity of samples prior to *ex vivo* analysis significantly affects the reproducibility and reliability of research outcomes [86]. Therefore, cell sequencing must be performed within a few hours after the sample is removed from the body; otherwise, the RNA phenotype of the cell will change significantly. Therefore, it is necessary to adapt high-performance cell freezing technology or cell RNA fixation technology to ensure the consistency of *ex vivo* body fluids and tissues with those *in vivo*. Presently, many automated single-cell isolation techniques depend on costly and large fluorescent flow cytometers. However, future advancements may enable rapid and immediate detection through the integration of microfluidic technology with high-throughput small sequencers. Consequently, future research should prioritize enhancing the efficiency of single-cell sorting, accelerating sequencing processes, reducing associated costs, and advancing data analysis tools. These improvements are essential to facilitate the broader implementation of scRNA-seq in clinical settings. If scRNA-seq is applied to clinical diagnosis and treatment, it is necessary to convert complex scRNA-seq data into clear, concise, and clinically valuable reports. Although whole exome sequencing has been widely used in tumor treatment, there is still a lack of relevant guidelines for the application of scRNA-seq to evaluate the efficacy of immunotherapy in patients.

Finally, considering the complexity involved in forecasting the efficacy of tumor immunotherapy, alongside the technical challenges associated with integrating multimodal data, artificial intelligence-driven computer modeling and machine learning could serve as advantageous catalysts for the clinical implementation of emerging technologies, such as scRNA-seq.

**Summary Points**

- Single-cell RNA sequencing (scRNA-seq) has significantly improved the ability to predict the efficacy of immune checkpoint blockade therapy.
- scRNA-seq is a valuable tool for facilitating the analysis of interactions between biomaterials and biological systems (e.g., tumor microenvironment) and would benefit the development of functional biomaterials for enhanced cancer treatment.
- In the future, effectively combining AI with multimodal evaluation models, such as scRNA-seq data, multi-omics, and tumor imaging, could enhance cancer prognosis and enable precision treatment. However, challenges remain, including data quality and availability, tumor heterogeneity versus model generalization, ‘black box’ issues versus clinical interpretability, integration of clinical complexity, lack of dynamic adaptability, and ethical and legal ambiguities.

## Abbreviations

AgNPs: silver nanoparticles
BCR: B-cell receptor
CAFs: cancer-associated fibroblasts
CAR-T: chimeric antigen receptor T-cell immunotherapy
CCR6: C-C motif chemokine receptor 6
CTLA-4: cytotoxic T-lymphocyte-associated protein 4
FOXP3: forkhead box P3
GNPs: garlic-derived nanoparticles
ICB: immune checkpoint blockade
IFN-γ: interferon-γ
IL-8: interleukin-8
IL-21: interleukin-21
LAG-3: lymphocyte activating 3
LAMP3: lysosomal-associated membrane protein 3
LBCLs: large B cell lymphomas
MHC: major histocompatibility complex
MNPs: magnetic nanoparticles
MPI: magnetic particle imaging
MRI: magnetic resonance imaging
MSI-H: microsatellite instability-high
NSCLC: non-small cell lung cancer
OS: overall survival
PD-1: programmed cell death receptor 1
PD-L1: programmed cell death ligand 1
4PD-1^hi^: PD-1-expressing CD4^+^FOXP3^-^ T cells
PET/CT: positron emission tomography/computed tomography
PIGR: polymeric immunoglobulin receptor
RTM: issue-resident macrophages
STAT1: signal transducer and activator of transcription 1
STAT3: signal transducer and activator of transcription 2
TAMs: tumor-associated macrophages
TCF7: transcription factor 7
TCR: T-cell receptor
TIBs: tumor-infiltrating B cells
TIMs: tumor-infiltrating myeloid cells
TLS: tertiary lymphoid structures
TMB: tumor mutation burden
TME: tumor microenvironment
TNF-α: tumor necrosis factor α
TRIM21: tripartite motif containing 21
Tregs: regulatory T cells
UMI: Unique Molecular Identifier
cDCs: conventional dendritic cells
cDNA: complementary DNA
dIgA: dimeric IgA
pHLIP: pH low insertion peptides
scRNA-seq: single-cell RNA sequencing
